# Spatial Positioning of All 24 Chromosomes in the Lymphocytes of Six Subjects: Evidence of Reproducible Positioning and Spatial Repositioning following DNA Damage with Hydrogen Peroxide and Ultraviolet B

**DOI:** 10.1371/journal.pone.0118886

**Published:** 2015-03-10

**Authors:** Dimitrios Ioannou, Lakshmi Kandukuri, Ameer Quadri, Victor Becerra, Joe Leigh Simpson, Helen G. Tempest

**Affiliations:** 1 Department of Human and Molecular Genetics, Herbert Wertheim College of Medicine, Florida International University, Miami, Florida, United States of America; 2 March of Dimes Foundation, White Plains, New York, United States of America; 3 Biomolecular Sciences Institute, Florida International University, Miami, Florida, United States of America; University of Oklahoma, UNITED STATES

## Abstract

The higher-order organization of chromatin is well-established, with chromosomes occupying distinct positions within the interphase nucleus. Chromatin is susceptible to, and constantly assaulted by both endogenous and exogenous threats. However, the effects of DNA damage on the spatial topology of chromosomes are hitherto, poorly understood. This study investigates the organization of all 24 human chromosomes in lymphocytes from six individuals prior to- and following in-vitro exposure to genotoxic agents: hydrogen peroxide and ultraviolet B. This study is the first to report reproducible distinct hierarchical radial organization of chromosomes with little inter-individual differences between subjects. Perturbed nuclear organization was observed following genotoxic exposure for both agents; however a greater effect was observed for hydrogen peroxide including: 1) More peripheral radial organization; 2) Alterations in the global distribution of chromosomes; and 3) More events of chromosome repositioning (18 events involving 10 chromosomes vs. 11 events involving 9 chromosomes for hydrogen peroxide and ultraviolet B respectively). Evidence is provided of chromosome repositioning and altered nuclear organization following in-vitro exposure to genotoxic agents, with notable differences observed between the two investigated agents. Repositioning of chromosomes following genotoxicity involved recurrent chromosomes and is most likely part of the genomes inherent response to DNA damage. The variances in nuclear organization observed between the two agents likely reflects differences in mobility and/or decondensation of chromatin as a result of differences in the type of DNA damage induced, chromatin regions targeted, and DNA repair mechanisms.

## Introduction

The nucleus is a highly complex and compartmentalized organelle that accommodates a wide spectrum of actions including: genome replication, transcription, splicing and DNA repair. The concept of nuclear organization can be considered with regards to chromatin only (i.e. position of chromosomes), the interchromatin compartment (channels around chromosome territories) and the specialized structures of the nucleus (nucleolus, nuclear matrix). Although the higher order of chromatin structure has been described extensively, the dynamics that govern the organization of chromatin, the underlying functional significance and the molecular mechanisms of relative change in position of chromosomes remain poorly understood.

Certain concepts have nonetheless emerged, with the central dogma of the field being chromosomes occupy distinct non-random positions within the interphase nucleus, these positions are termed chromosome territories (CTs) [1–6]. CTs appear to be highly partitioned with minimal interactions which seems to confer a fractal globule model for the genome at least on the megabase scale as shown by 3C studies [7]. This minimal interaction occurs exclusively in the periphery of CTs, is observed in higher eukaryotes and contrasts the situation in budding yeast, where CTs are less geographically defined and are characterized by a higher degree of intermingling [8]. Identification of patterns of proximity (i.e. radial organization) among chromosomes may have provided a functional advantage over the course of evolution. Two models have emerged to describe the radial organization of CTs within the interphase nucleus, namely gene density and chromosome size.

The gene density model stems from observations in proliferating lymphoblasts and fibroblasts that gene rich chromosomes are located toward the nuclear interior with gene poor chromosomes located toward the nuclear periphery [9,10]. The gene density model has also been observed in primates, [11], old world monkeys [12], rodents [13], cattle [14] and chicken (however, the chicken also fits the chromosome size model) [15]. The chromosome size model suggests CTs are ordered according to size, with small chromosomes preferentially localized toward the nuclear interior and larger chromosomes toward the nuclear periphery. This model was proposed following observations in quiescent and senescent cells [16,17] and also 3D-FISH experiments in flat ellipsoid fibroblasts [18]. The two prevailing models should not be considered as mutually exclusive, given that chromosome position likely depends on the proliferating status of the cell, the chromosome and/or its neighborhood [17,19]. These correlative observations have established the concept of non-random position of chromosomes in the interphase nucleus and have raised the question of the functional significance of this organization. One prevailing hypothesis correlates gene activity with an interior localization. Several lines of evidence lend support for this hypothesis given that the following have all been observed to be localized towards the nuclear interior: 1) Gene rich chromosomes; 2) G-C rich regions of chromatin; and 3) Early replicating regions of the genome, which typically contains active genes [20]. Further support for a possible regulation of gene expression from the nuclear “address” of chromosomes comes from experiments where CTs are reorganized upon a surge of transcription [21] during cellular differentiation processes (e.g. β-globin genes in mouse erythroid cells, genes during adipogenesis). In such instances activated genes have been demonstrated to reposition from the nuclear periphery to the nuclear interior [20,22]. In addition, recent observations demonstrating relocation of activated genes with nuclear structures involved in transcription (e.g. RNA polymerase II molecules and Cajal bodies) provides further support for this hypothesis [21]. Whether genome organization determines function or whether localization is a “reflection” of function continues to be debated. However, several key findings denote its importance in maintaining a stable architecture for proper cellular functionality [23]. These include: 1) Non-random organization of chromosome position in multiple cell types and evolutionarily divergent species [6]; 2) Evidence that this pattern of organization is evolutionary conserved [24]; and, 3) Indications that alterations in nuclear localization are correlated with certain diseases (laminopathies, Hutchinson-Gilford Progeria, Promyelotic leukemia, and breast cancer) [25–27]. Any perturbation in nuclear architecture could thus induce change in the local gene environment and availability of transcription factors leading to possible misregulation or failure to take part in transcription [28]. Thus, it seems reasonable to suggest that the nucleus requires a “healthy” state of organization for proper functionality, and if this state is perturbed it could be manifested as alteration of chromosome (and thus gene) position.

Genomic DNA is constantly under attack from endogenous and exogenous factors such as reactive oxygen species (ROS) arising from normal cellular metabolism or physical and chemical agents such as ultraviolet (UV) radiation, alkylating agents and topoisomerase inhibitors [29]. To maintain genomic integrity from the detrimental effects of damaging agents (e.g. mutations and chromosomal rearrangements) numerous repair mechanisms have evolved. These various mechanisms comprise the DNA damage response (DDR) that either work independently or in combination to repair damaged lesions and allow the cells to re-enter the cell cycle for faithful duplication of the genome [30]. This study has focused on genotoxic agents namely, hydrogen peroxide (H_2_O_2_) and ultraviolet (UV) radiation that are capable of inducing endogenous and exogenous DNA damage.

H_2_O_2_ is a by-product produced by ROS during normal cellular metabolic activity that can induce single- and double-stranded breaks (SSBs & DSBs), helical distortions and hindrances to base pairing. These mechanisms can alter important genetic information by interfering with replication and transcription. Thus, accumulation of oxidative lesions compromises DNA integrity predisposing to cancer and aging [31]. UV radiation is a common exogenous agent, with UVC (100–280nm) mostly absorbed by the earth’s atmosphere, whereas UVA (315–400nm) and UVB (280–315nm) reach the earth’s surface and are known to cause mutagenic and cytotoxic lesions within DNA [32]. UVA causes oxidative damage, whereas UVB causes dipyrimidine photoproducts (thymine dimers) by a direct photochemical mechanism [33]. The most common DNA repair mechanism to respond to oxidative damage by H_2_O_2_ occurs through the activation of inherent antioxidant enzymes possessed by cells [34] whereas, nucleotide excision repair (NER) is the predominant mechanism that repairs damage caused by UV radiation [35].

The compartmentalization of the nucleus with the radial non-random organization of chromosomes seems to have a modulatory role for induction of DNA damage. Therefore, if DNA damage preferentially occurs in certain regions of the nucleus the radial organization of chromatin will be impacted with specific chromosomes more prone to DNA damage. The bodyguard hypothesis proposes that peripherally localized heterochromatin protects the interior part of the cell [36]. However, evidence has been provided to suggest that the nuclear center (location of gene dense chromosomes in lymphocytes) could be the preferred site for DNA damage and mutation [37]. Further evidence for the generation of preferential sites of DNA damage is provided by the formation of recurrent chromosomal translocations; and the recruitment of repair mechanisms [37] at different rates depending on the chromatin type. Typically, gene rich euchromatic regions are repaired at a faster rate than gene poor, heterochromatic regions [38].

Perturbations in the nuclear address of CTs following induction of DNA damage can provide unique insights into chromatin behavior following damage on a global scale and can potentially provide more details into specific cell type patterns of response to damage. In the current study we assessed the radial topology of all 24 human chromosomes in lymphocytes collected from six healthy volunteers. DNA damage was induced in-vitro in lymphocytes utilizing two different genotoxic agents that differ in their mechanistic action (H_2_O_2_ and UVB). Assessment of the radial organization of all 24 chromosomes before and after in-vitro exposure to the genotoxic agents was studied in all six individuals. Our study findings of inter-individual reproducibility in CT organization and genotoxic specific alterations in topology following exposure to H_2_O_2_ and UVB will be discussed.

## Materials and Methods

### Sample cohort

This research study was approved by the Florida International University Institutional Review Board (IRB). Informed written consent to participate in this study was provided by six individuals (four females and two males). The average age of the participants was 29.8 years (range, 20–40). As per the IRB protocol each participant filled out a brief health history survey, providing life style (e.g. alcohol or tobacco use) any recent illness information, and any medication taken. All participants were non-smokers, had not knowingly been in contact with any hazardous or radioactive material in their working or home environment. Four subjects were social drinkers (2–3 units per week) and only one participant disclosed a medical condition (hyperthyroidism) for which eltroxin was prescribed.

### Cell culture conditions and genotoxic exposure

Peripheral blood was collected by venipuncture in heparin tubes (Greiner-BioOne, Monroe, NC, USA). Whole blood from each individual was split and cultured in the presence or absence of genotoxic agents. Unexposed “control” lymphocyte cultures were prepared as follows: Culture medium RPMI 1640 (Lonza, Walkersville, MD, USA) was reconstituted with 10% heat inactivated fetal bovine serum (FBS—Sigma-Aldrich, St Louis, MO, USA), 2% L-glutamine (Thermo-Fisher, Waltham, MA, USA) and 1% penicillin-streptomycin solution (Thermo-Fisher, Waltham, MA, USA). All cultures had a total volume of 5ml of reconstituted medium with 100μl of phytohaemagglutinin (PHA), (45mg/vial) (Remel Inc, Lenexa, KS, USA) 0.8–1.0 ml of blood was incubated for 71 hours at 37°C (5% CO_2_) to allow mitotic proliferation of lymphocytes.

To induce DNA damage, lymphocyte cultures were exposed at the initiation of culturing to H_2_O_2_ (80mM—30 minutes) (Thermo-Fisher, Waltham, MA, USA) or UVB radiation (280–320nm—15 minutes), from a BIO-RAD trans-illuminator (BIO-RAD, Hercules, CA, USA). The maximal concentration of H_2_O_2_ and UV exposure were chosen based upon previous dose-response experiments. The genotoxity of a range of different conditions for both genotoxic agents were tested to determine which conditions resulted in presence of measurable DNA damage after 71 hours of culturing following exposure. Maximal doses of the genotoxic agents were also selected to ensure that following exposure, a high proportion of cells were still viable and capable of undergoing cell division. Specific treatment conditions (80mM for H_2_O_2_ and 15 minutes for UVB) were selected based on the presence of a minimum of 25 metaphase spreads for karyotype analysis, detectable chromosome aberrations, and a reduction in mitotic index that did not exceed 65%. Karyotyping was used to identify whether the genotoxic agents utilized had clastogenic or aneugenic properties detected by the presence of cytogenetically visible structural or numerical chromosomal aberrations. In addition, the mitotic index was assessed to provide a measure of the proliferation status of the cell culture population. The mitotic index is a ratio between the number of cells in mitosis (complete metaphases) and total number of cells.

The H_2_O_2_ exposed cultures were incubated in the presence of 80mM H_2_O_2_ for 30 minutes at 37°C in complete medium without PHA, cultures were subsequently centrifuged at 1,200 rpm for 10 minutes to halt the reaction, the supernatant was removed and cells were resuspended in 5ml of complete medium with PHA for incubation (71 hours) as outlined for the unexposed cultures. Additionally, lymphocyte cultures (in complete medium with PHA) were also exposed to UVB radiation (280–320nm) utilizing a BIO-RAD trans-illuminator (BIO-RAD, Hercules, CA, USA) for 15 minutes at room temperature prior to being transferred at 37°C for incubation (71 hours).

### Karyotyping and calculation of the mitotic index

Evaluation of the DNA damage induced by the genotoxic agents (H_2_O_2_ or UVB) at the chromosomal level was performed using standardized cytogenetic procedures. In brief, lymphocytes were cultured in the absence of genotoxic agents for 71 hours, subsequently, proliferating cells in metaphase were arrested using 0.2μg colcemid (Thermo-Fisher, Waltham, MA, USA) for 30 minutes at 37°C, followed by standard hypotonic conditions to allow separation of white blood cells from anucleate erythrocytes (0.075M of KCL—Thermo-Fisher, Waltham, MA, USA) for 45 minutes at 37°C. White blood cells were subsequently fixed in 3:1 (v/v) of methanol:acetic acid solution to clean and fix the preparation. All cultures were stored at -20°C immediately following the harvesting procedure. Cells were dropped on glass slides (FisherBrand—Thermo-Fisher, Waltham, MA, USA) and mounted with a glycerol-based solution containing 4′,6-diamidino-2-phenylindole (DAPI) (Vectashield with DAPI—Vector Labs, Burlingame, CA, USA) under a 24X55 mm coverslip. Metaphases were captured using an Olympus BX61 epifluorescence microscope equipped with a cool charged couple device camera (Hamamatsu ORCA—R_2_ C10600). All images were acquired using Smart Capture 3.0 and chromosomal analysis was performed using Smart Type 2.0 (Digital Scientific, Cambridge, UK). Karyotyping was performed using reverse DAPI staining to visualize chromosome banding. Chromosomes and chromosome aberrations were identified and described using the standardized International System for Human Cytogenetic Nomenclature [39]. A minimum of 25 metaphase spreads were karyotyped per subject, per condition, when possible at least 50 metaphase spreads were karyotyped. The mitotic index was calculated by analyzing a minimum of ten fields of view and scoring a minimum of 1000 cells respectively for each condition. The mitotic index of cultures exposed to genotoxic agents was subsequently compared to unexposed cultures to determine the percentage change in cellular proliferation.

### Fluorescence in situ hybridization (FISH)

Cells (from unexposed, H_2_O_2_, and UVB exposed cultures) were dropped on glass slides, allowed to adhere by ageing overnight at room temperature (RT) and then washed in 1X PBS (Thermo-Fisher, Waltham, MA, USA), followed by an ethanol dehydration step (70–80–100% for 3 minutes each). Air dried cells were then treated with 1% pepsin solution (Thermo-Fisher, Waltham, MA, USA) in a pre-warmed at 37°C solution of 49 ml double distilled water (ddH_2_O) and 0.5 ml of 1N HCL (Thermo-Fisher, Waltham, MA, USA) for 20 minutes. Cells were then rinsed with ddH_2_O and 1 X PBS at RT, and subjected to another round of fixation using 1% paraformaldehyde/PBS [1.34ml of 37% paraformaldehyde (Thermo-Fisher, Waltham, MA, USA) in 49 ml of PBS] at 4°C for 10 minutes. Slides were then rinsed in 1 X PBS followed by ddH_2_O at RT, in preparation for another dehydration round in ethanol (2 minutes each), and finally air dried. A dual color FISH experiment (red and green FISH probes) was then set up utilizing probes for whole chromosome paints (WCPs), for all 24 chromosomes. All probes were obtained from Rainbow Scientific (Windsor, CT, USA) and were co-denatured for 5 minutes with lymphocytes at 75°C followed by overnight hybridization (>16hours) at 37°C using a Thermobrite Statspin (Abbott Molecular, Illinois, IL, USA). A post hybridization stringency wash was performed in a pre-warmed 73°C solution of 0.7 X SSC/0.3% Tween 20 (Thermo-Fisher, Waltham, MA, USA) (35ml of 20 X SSC, 3ml of Tween 20 and 965ml of ddH_2_O) for 2 minutes. After 2 minutes elapsed, cells were washed in 2 X SSC/ 0.1% Tween 20 (100ml of 20 X SSC, 1ml of Tween 20 and 900ml of ddH_2_O) and a brief ethanol series (1 minute each). Slides were subsequently air dried in the dark and mounted with DAPI under a 24X55mm coverslip. Image acquisition was performed as described above using 3 single band pass filters for (fluorescein isothiocyanate (FITC), tetramethyl rhodamine isothiocyanate (TRITC), and DAPI) (Chroma Technology, Bellows Falls, VT, USA). All images were acquired using Smart Capture 3.0, exported as. tiff files for further analysis. Captured images were subjected to judicious thresholding to reduce any background fluorescence present, extreme care was taken to ensure the intensity and distribution of the FISH probe signal within the nucleus was not compromised or altered. A minimum of 100 cells were analyzed per subject, per chromosome pair, per condition (unexposed, H_2_O_2_ and UVB exposed).

### Radial chromosome positioning analysis

To evaluate the chromosome position and therefore the nuclear organization, previously published methodologies were utilized [9,10]. The details have been described extensively elsewhere [40,41]. Briefly, a customized script was written for Image J, which allows for the separation of each image into three channels (red and green [FISH probes] and blue [DAPI counterstain]). The DAPI fluorescence is converted to a binary mask that allows for the creation of 5 rings of equal area (1- interior, 5- peripheral). The proportion of WCP signal in each ring (and for each channel) is measured relative to the total signal for the area that is covered by the ring. Data is collected and normalized against the different DNA content (DAPI fluorescence intensity) in the nucleus to compensate for the fact that a 3D object is observed under two-dimensions. The radial distribution of a CT is analyzed across the population of nuclei (n = 100). Our method of analysis also allows the data to be transformed to provide a single number for each nucleus reflective of the overall position of the signal. The software compresses the entire CT distribution across the five rings by weighing the proportion of signal contained within each ring and summing these together across the whole population of cells analyzed. For example if we consider a single cell in which a CT was entirely localized in rings 2 and 3 with an equal proportion of fluorescence signal within both rings, the software would weight this distribution as follows: 2*0.5+3*0.5 = 2.5. In doing so, we are able to compress the relatively large CTs of the population of cells analyzed (n = 100/CT/subject) into a single number, which reflects the midpoint of the frequency distribution of observed fluorescence (median). This median can be utilized to determine the hierarchical radial order of CTs from the nuclear interior toward the nuclear periphery.

### Statistical analysis

Chi-squared goodness-of-fit test (χ^2^) was employed to compare the distribution of each individual CT across each of the five rings for each cell and individual. If the distribution of the CT was equally distributed across the five rings, the CT was classified as random (p>0.05). Non-random organization of a CT was supported if the CT distribution was not equally distributed across the five rings (p<0.05). The chi-squared goodness-of-fit test (χ^2^) was applied to determine subject specific differences in CT organization. In this instance the distribution of fluorescence within the five rings for each CT was compared between subjects, (p>0.05 suggested a reproducible CT distribution between subjects, whereas p<0.05 provided evidence of a different CT distribution between subjects). Additionally, the chi-squared goodness-of-fit test (χ^2^) was used to evaluate whether CT repositioning occurred in the H_2_O_2_ and UVB exposed cultures by comparing the CT distribution in the unexposed culture from the same individual, (p>0.05 provided evidence of no change in CT distribution following exposure, whereas p<0.05 provided evidence of altered CT positioning).

## Results

### Measurement of genotoxicity, karyotyping and mitotic index assessment

**Fig 1:**
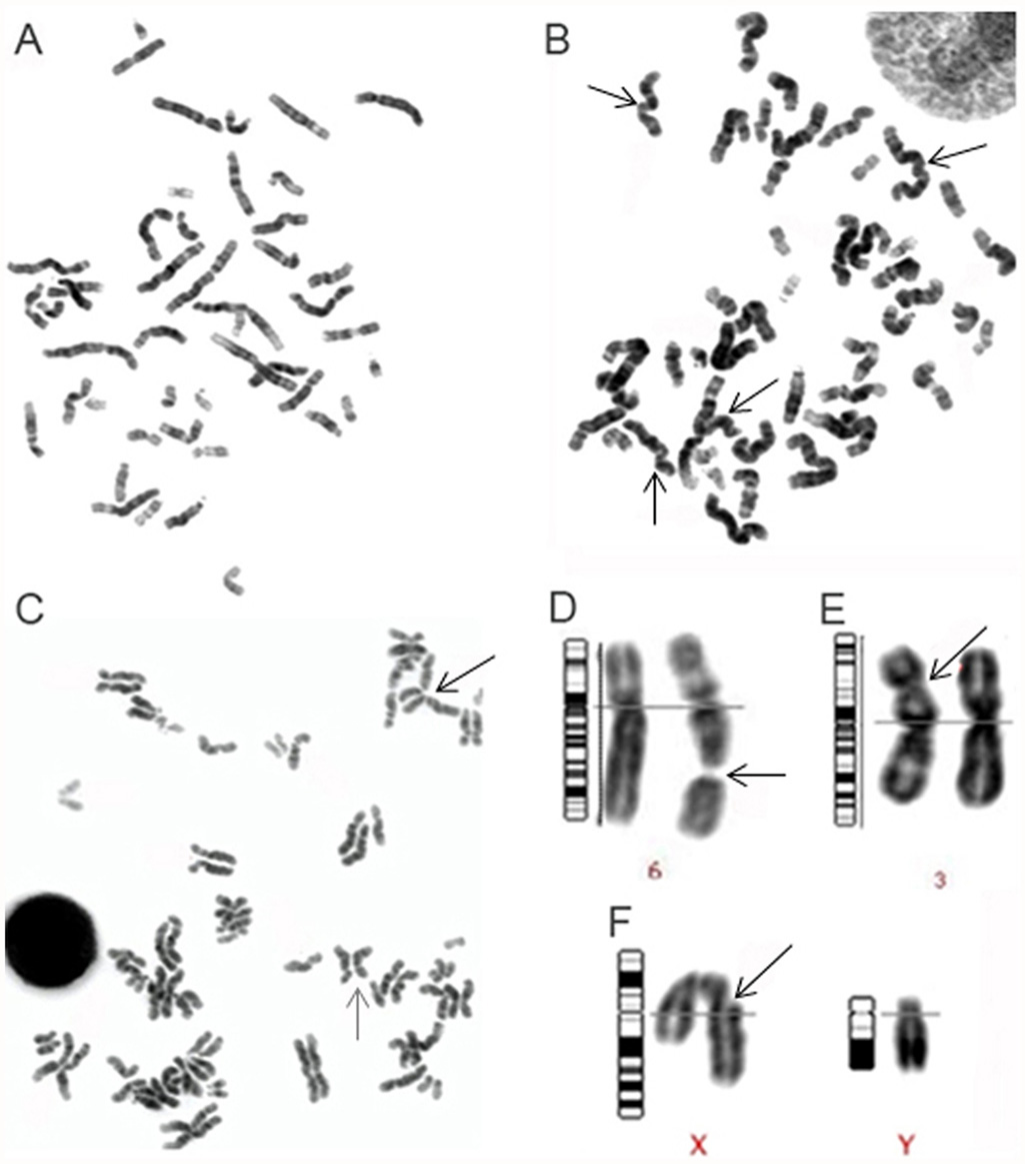
Chromosomal aberrations observed following exposure to genotoxic agents. Panels A-F provides representative examples of karyotypes and different chromosomal aberrations observed after genotoxic exposure. Panels A-C depict individual metaphase spreads. Panels D-F provides examples of individual chromosome pairs with specific chromosome aberrations (magnified). A description of each panel follows: (A) Normal 46, XX metaphase spread; (B) Metaphase spread displaying distorted pulverized chromosomes with altered morphology, indistinct banding pattern and indistinguishable centromeres (several examples are indicated by arrows); (C) Metaphase spread demonstrating premature sister-chromatid separation (e.g. black arrow) and premature centromere separation (e.g. gray arrow); (D) Gap in chromosome 6; (E) Dicentric chromosome 3 at band 3p21; and (F) Chromatid break in the X chromosome at band Xq21.

Peripheral blood from six individuals was cultured in the presence and absence of genotoxic agents. To assay the genotoxicity of the tested agents, metaphase spreads from each individual and experimental condition were karyotyped and the mitotic index was calculated. A minimum of 50 metaphases were karyotyped from the unexposed cultures for each of the six individuals. A notable decrease in the number of metaphase spreads available for karyotyping was observed in the H_2_O_2_ and UVB exposed cultures, with an average of 37.8 and 33.3 metaphases karyotyped, per subject, respectively. Further evidence of a reduction in cellular proliferation was provided by evaluating the mitotic index. Compared to the unexposed lymphocyte cultures there was an average of a 62% and 40.5% reduction in cellular proliferation in H_2_O_2_ and UVB treated cells respectively. These findings were most likely due to altered cell cycling of lymphocytes exposed to genotoxic agents resulting in a significant reduction in cell proliferation. Over 300 metaphases were karyotyped for the unexposed lymphocytes, all metaphases were cytogenetically normal with the exception of one metaphase spread that contained a single chromatid break. The karyotyping analysis for the exposed conditions in all six subjects, revealed cytogenetically visible chromosome aberrations in 8.8% and 8% of H_2_O_2_ and UVB treated metaphases respectively (Fig. 1). The most common aberration observed in both conditions was pulverized chromosomes, the morphology of the chromosomes being highly distorted. Pulverization of the chromosomes occurred in 14 H_2_O_2_ metaphases and six UVB metaphases. The second most commonly observed aberration was chromosome or chromatid breaks that occurred in four H_2_O_2_ metaphase spreads and six UVB metaphases. In both H_2_O_2_ and UVB metaphases one metaphase spread was found to possess premature centromere separation and premature separation of sister chromatids (Fig. 1). The remaining aberration identified in H_2_O_2_ cells was the presence of a dicentric chromosome. In the UVB exposed samples the following additional aberrations were identified: two chromatid gaps, one chromosome gap, one numerical aberration (47, XYY), and one marker chromosome.

### Radial organization of all 24 human chromosomes in unexposed and exposed lymphocytes

**Fig 2:**
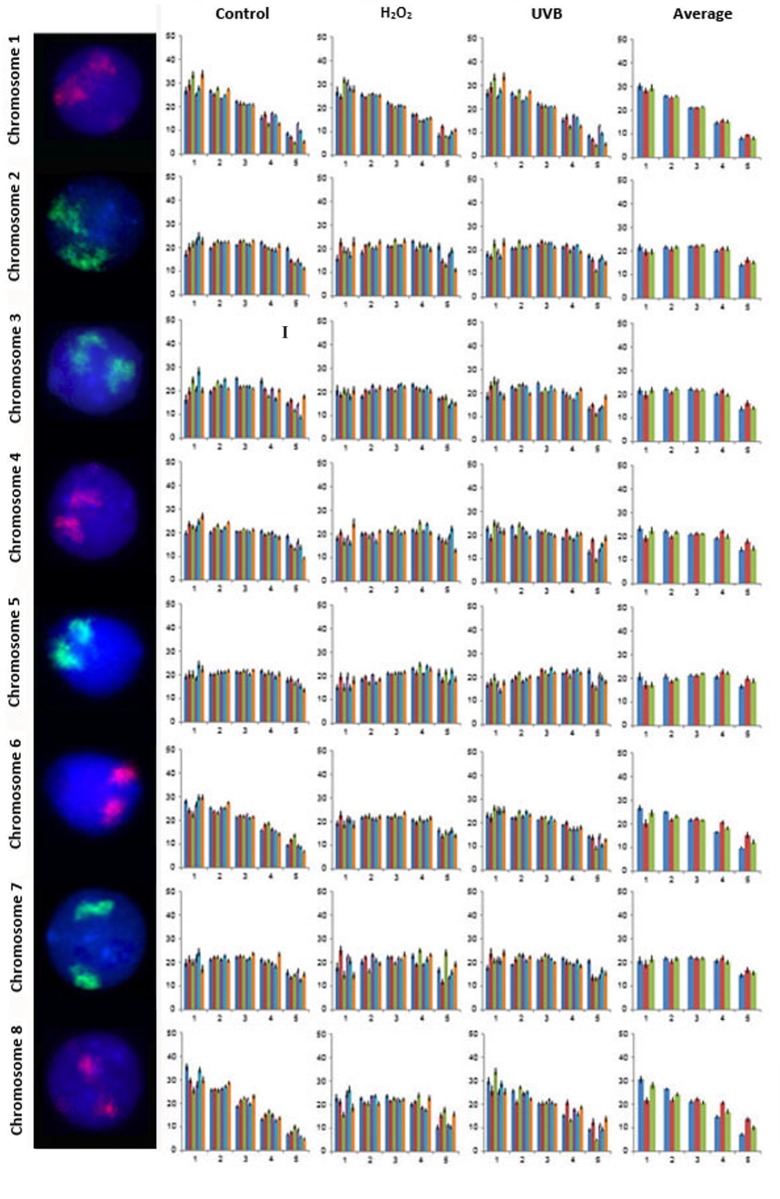
Radial distribution for chromosomes 1–8 in six subjects in unexposed, H_2_O_2_ and UVB exposed lymphocytes. Fig. 2 displays representative FISH images and the radial distribution for CTs 1–8. Note FISH experiments were dual color, for simplicity only a single fluorochrome (CT) is displayed in each lymphocyte (the second fluorochome was removed by deselecting either the red or green channel). Moving from left to right the chromosome number is indicated followed by a representative FISH image for the CT and four histograms. The X-axis for all histograms represents each of the five rings of equal area (1–5, nuclear interior to nuclear periphery [left to right]). The Y-axis for all histograms represents the proportion of fluorescence (%). Error bars represent the standard error of the mean (SEM). The first, second and third histogram display the radial distribution for each CT in control, H_2_O_2_ and UVB exposed lymphocytes, respectively. Each of these histograms contain six bars for the five rings, corresponding to each of the six subjects (1 to 6, left to right). The fourth histogram displays the average radial distribution for the six subjects in unexposed (blue), H_2_O_2_ (red) and UVB (green) exposed lymphocytes. Roman numerals indicate significant inter-individual variations in radial distributions (p<0.05) between subjects in control lymphocytes: I- CT3 subject 5 (different to subjects 1 and 6).

**Fig 3:**
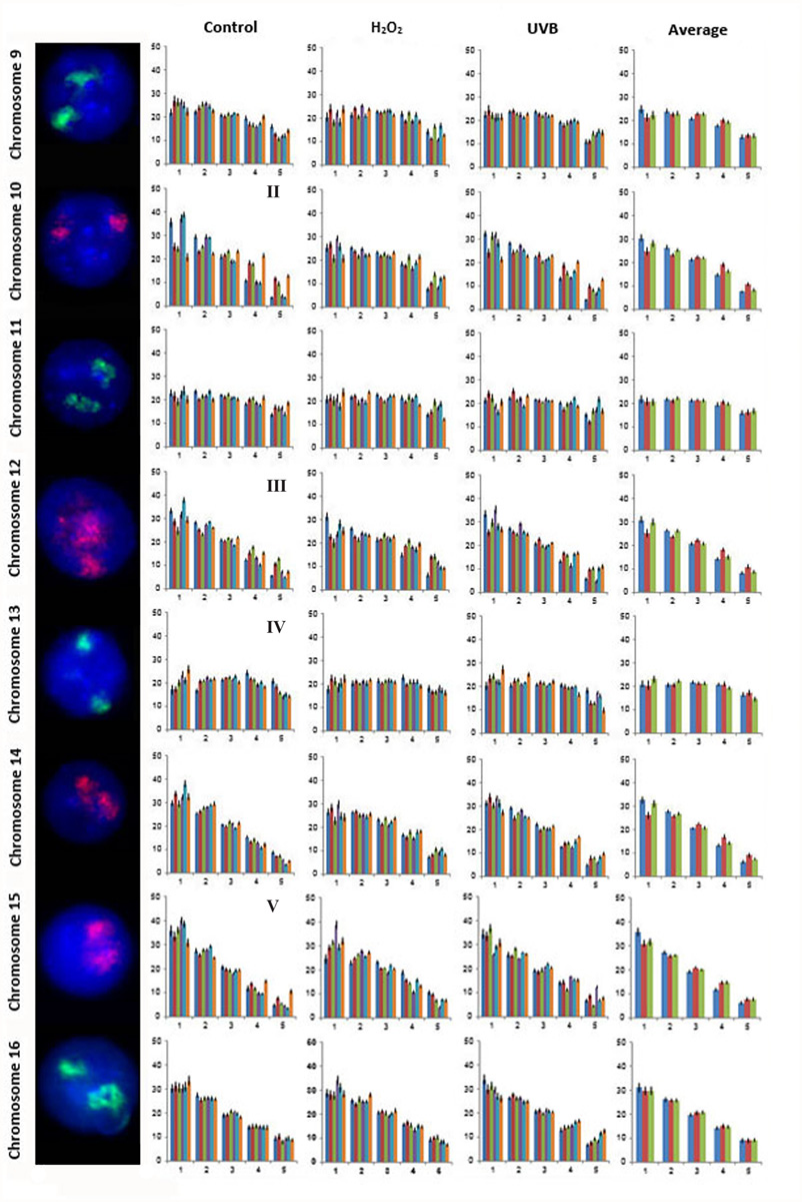
Radial distribution for chromosomes 9–16 in six subjects in unexposed, H_2_O_2_ and UVB exposed lymphocytes. Fig. 3 displays representative FISH images and the radial distribution for CTs 9–16. As in Fig. 2 moving from left to right the chromosome number is indicated followed by a representative FISH image for the CT and four histograms. Each histogram displays the proportion of fluorescence (%) from the nuclear interior toward the nuclear periphery (left to right). The first, second and third histogram displays the radial distribution for each CT in control, H_2_O_2_ and UVB exposed lymphocytes, respectively for each of the six subjects (1 to 6, left to right). The fourth histogram displays the average radial distribution for the six subjects in unexposed (blue), H_2_O_2_ (red) and UVB (green) exposed lymphocytes. Error bars represent the standard error of the mean (SEM). Roman numerals indicate significant inter-individual variations in radial distributions (p<0.05) between subjects in control lymphocytes: II- CT10 subjects 1, 4 and 5 (different to subjects 2, 3 and 6); III- CT12 subject 3 (different to subjects 1 and 5); IV- CT13 subject 6 (different to subject 1); V- CT15 subject 6 (different to subject 4).

**Fig 4:**
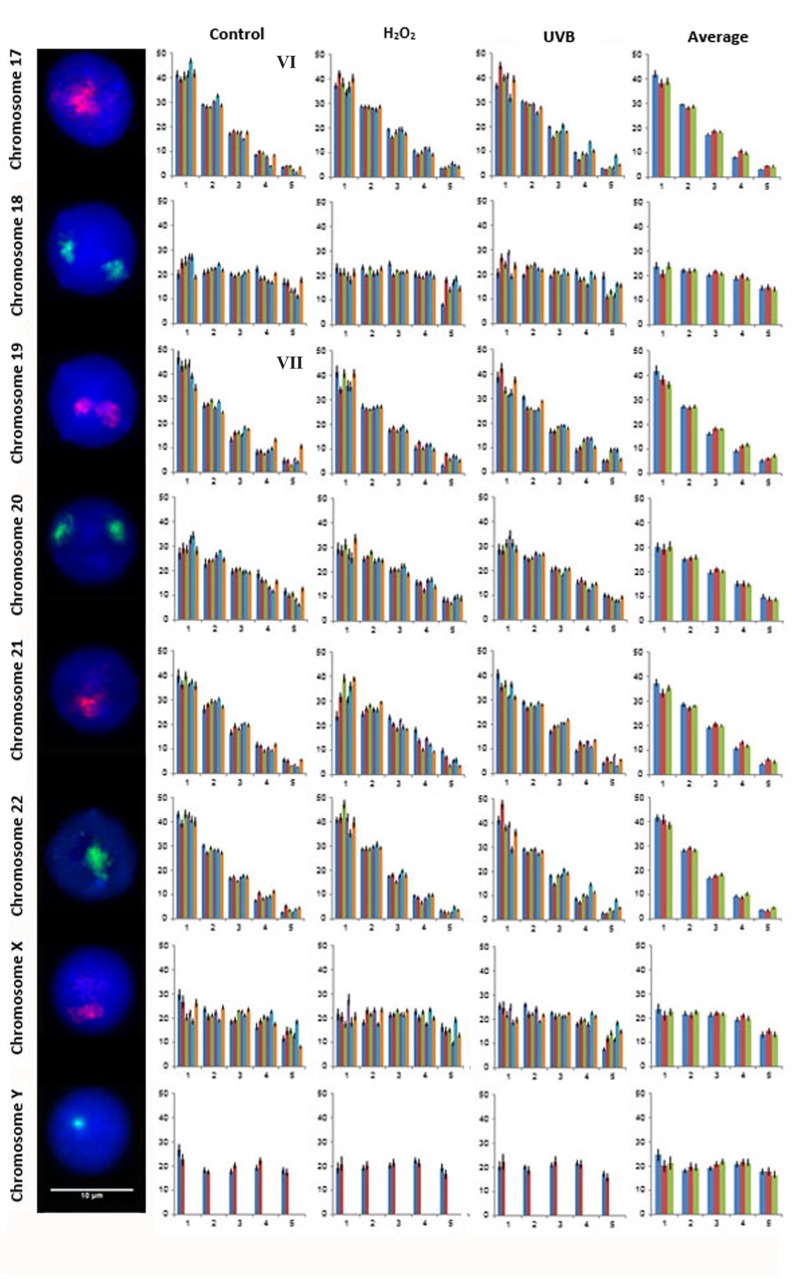
Radial distribution for chromosomes 17–22, X and Y in six subjects in unexposed, H_2_O_2_ and UVB exposed lymphocytes. Fig. 4 displays representative FISH images and the radial distribution for CTs 17–22, X and Y. As in Figs. 2 and 3 moving from left to right the chromosome number is indicated followed by a representative FISH image for the CT and four histograms. Each histogram displays the proportion of fluorescence (%) from the nuclear interior toward the nuclear periphery (left to right). The first, second and third histogram displays the radial distribution for each CT in control, H_2_O_2_ and UVB exposed lymphocytes, respectively for each of the six subjects (1 to 6, left to right), with the exception of CTY which only contains data from the two male subjects enrolled in this study (subjects 1 and 2). The fourth histogram displays the average radial distribution for the enrolled subjects in unexposed (blue), H_2_O_2_ (red) and UVB (green) exposed lymphocytes. Error bars represent the standard error of the mean (SEM). Roman numerals indicate significant inter-individual variations in radial distributions (p<0.05) between subjects in control lymphocytes: VI- CT17 subject 6 (different to subject 5); and VII- CT19 subject 6 (different to subjects 1, 2, 3, 4, and 5).

A total of 21,600 cells were captured and analyzed from all six subjects to assess the nuclear organization for all 24 human chromosomes in lymphocytes from control and genotoxicant exposed cultures. Examples of FISH images for the CTs, the radial distribution of all 24 CTs in the lymphocytes of each of the six subjects for each condition and the average distribution of all six subjects for each of the three conditions and is presented in Figs. 2–4 (CTs 1–8 Fig. 2; CTs 9–16 Fig. 3; and CTs 17–22, X and Y Fig. 4). One unique aspect of this study is the inclusion of multiple subjects which enables the reproducibility of CT organization for each chromosome within the same cell type to be investigated between subjects. In this study, the radial organization of CTs in the unexposed lymphocytes was assessed 140 times (chromosomes 1–22 and X for 6 subjects [n = 138], and the Y chromosome [n = 2] from the two male subjects enrolled in this study). The chi-squared goodness-of-fit comparison was utilized to examine inter-individual differences in the radial distribution of CTs. The radial organization was remarkably consistent between the six subjects in the unexposed lymphocytes with no significant differences identified in the radial distribution between subjects for the vast majority of CTs (1, 2, 4, 5, 6, 7, 8, 9, 11, 14, 16, 18, 20, 21, 22, X and Y [p>0.05]). However, there were 19/140 occasions (13.57%) of inter-individual variability in the CT radial distribution, involving only seven chromosomes (Figs. 2–4). Specifically, the following subjects showed differences in the radial distribution of CTs compared to other subjects: subject 5: CT3 compared to subjects 1 and 6, (p<0.05); CT10 subjects 1, 4 and 5 compared to subjects 2, 3 and 6 (p<0.05); subject 3: CT12 compared to subjects 1 and 5, (p<0.05); subject 6: CT13 compared to subject 1, CT15 compared to subject 4, CT17 compared to subject 5 and CT 19 compared to subjects 1, 2, 3, 4, and 5 (p<0.05).

**Fig 5:**
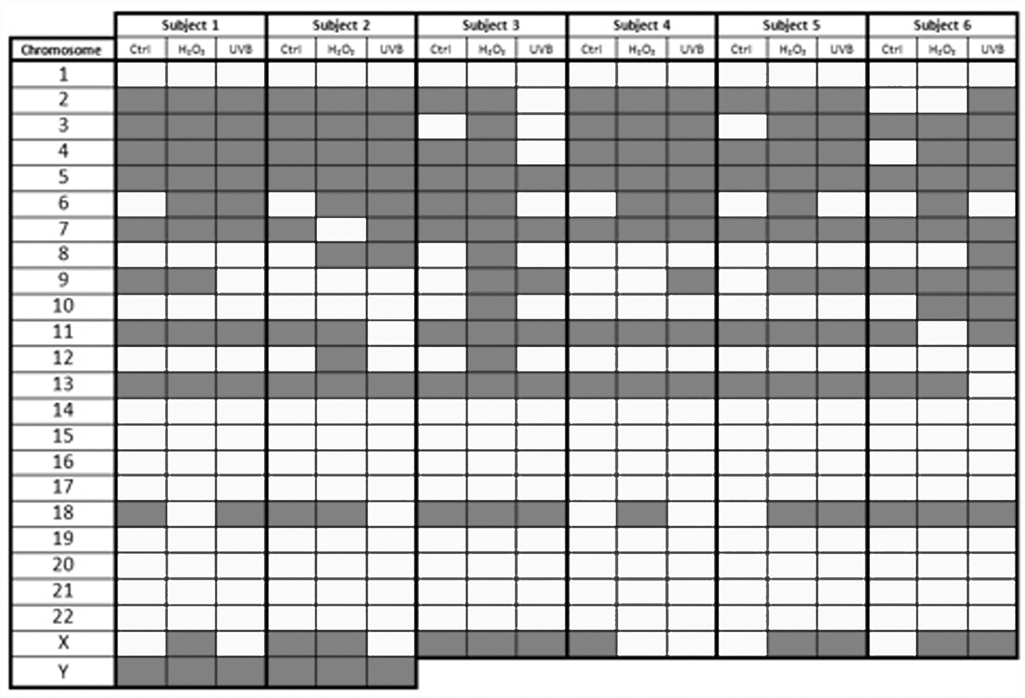
Non-random or random chromosome position status for all subjects and conditions. Each colored block represents the status of the chromosome position for all six subjects and three tested conditions following analysis of 100 cells. Chromosome territory position was determined to be random or non-random by the χ^2^ goodness of fit test (df:4). White blocks indicate non-random positioning (p<0.05), whereas grey blocks indicates random positioning. Data for chromosome Y is provided only for the two male subjects. The control (unexposed) condition is abbreviated in the table (Ctrl).

The random or non-random organization of the individual CTs for all six individuals for each of the treatment conditions was also assessed (Fig. 5). In unexposed lymphocytes the vast majority of CTs demonstrated consistent reproducible patterns of non-random/random organization in all six individuals. Chromosomes 1, 8, 10, 12, 14, 15, 16, 17, 19, 20, 21, and 22 were classified as non-randomly organized. In contrast, chromosomes 5, 7, 11, 13, and Y randomly organized in all six individuals. The remaining seven CTs (2, 3, 4, 6, 9, 18, and X) demonstrated more inter-individual variability in non-random/random status among the different subjects enrolled in this study. For example, chromosomes 6 and 18 were non-randomly organized in 5 out of 6 and 2 out of 6 subjects, respectively. In the H_2_O_2_ exposed lymphocytes the following chromosomes 1, 14, 15, 16, 17, 19, 20, 21, and 22 were non-randomly organized, whereas chromosomes 3, 4, 5, 6, 13, and Y were randomly organized in all six subjects. Nine CTs had inter-individual variability (chromosomes 2, 7, 8, 9, 10, 11, 12, 18, and X). However, four chromosomes demonstrating inter-individual variations in organization were common between the control and H_2_O_2_ treated cells (chromosomes 2, 9, 18, and X). In cultures treated with UVB, chromosomes 1, 12, 14, 15, 16, 17, 19, 20, 21, and 22 were non-randomly organized, whereas chromosomes 5, 7, and Y were randomly organized in all six subjects (Fig. 5). Eleven CTs depicted inter-individual variability (chromosomes 2, 3, 4, 6, 8, 9, 10, 11, 13, 18, and X); with seven chromosomes being common to either unexposed or H_2_O_2_ cultures (chromosomes 2, 3, 4, 6, 9, 18, X and 2, 8, 9, 10, 11, 18, and X, respectively). Interestingly chromosomes 2, 9, 18, and X were the only CTs with inter-individual variability for all conditions, with CTs 1, 14, 15, 16, 17, 19, 20, 21, and 22 demonstrating consistent non-random organization, and CT’s 5 and Y demonstrating consistent random organization in all individuals and all treatment conditions. The emerging picture from Fig. 5 is that a large proportion of chromosomes occupy distinct positions in all conditions and that their non-random/random status is largely reproducible among subjects. CTs from all subjects were non-randomly organized in 64.28% (90/140), 52.14% (73/140), and 60% (84/140) of unexposed H_2_O_2_ and UVB exposed samples respectively.

### Genotoxicity effect on radial CT organization

**Fig 6:**
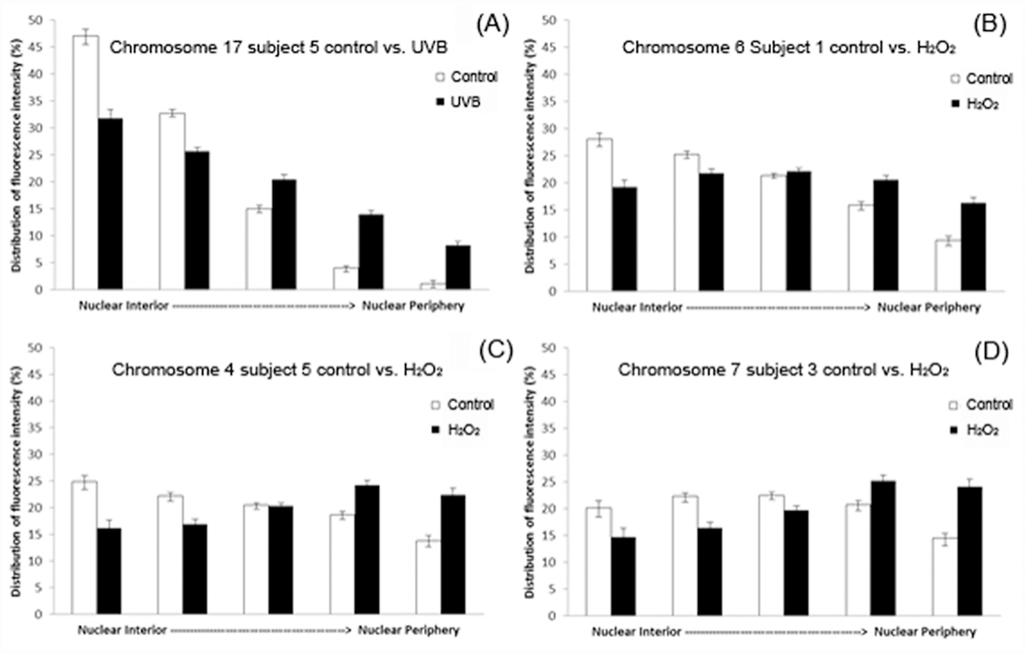
Chromosome repositioning events following exposure to genotoxic agents. All four panels (A-D) depict the different types of CT repositioning observed in human lymphocytes following exposure to genotoxic agents H_2_O_2_ and UVB compared to control (unexposed) cells. In all four panels the X axis shows the five shells of equal area from the nuclear interior to the nuclear periphery (left to right) and the Y axis shows the percentage of fluorescence for each CT that lies within each of the five shells after analysis of 100 cells. Error bars represent the standard error of the mean (SEM). Examples for each of the four different categories of statistically significant chromosome repositioning described in Table 1 are provided: (A) “less interior” localization of chromosome 17 in subject 5 following UVB exposure compared to control cells; (B) interior to intermediate positioning of chromosome 6 in subject 1 following H_2_O_2_ exposure; (C) interior to peripheral positioning of chromosome 4 in subject 5 following H_2_O_2_ exposure; and (D) intermediate to peripheral positioning of chromosome 7 in subject 3 following H_2_O_2_ exposure.

In order to evaluate statistically significant repositioning of CTs in control and exposed lymphocytes the distribution of fluorescence in each of the five shells of equal area were compared (between the control and H_2_O_2_/ UVB exposed lymphocytes from the same individual). When the p value from the chi-squared goodness-of-fit comparison was less than 0.05 the topological alteration was deemed statistically significant. Table 1 provides a summary of the changes that were observed in CT localization based on these comparisons. Certain interesting inferences can be drawn from Table 1. Cumulatively there were 29 events of repositioning following exposure of lymphocytes to H_2_O_2_ and UVB (18 and 11 events of repositioning, respectively). Furthermore, all subjects participating had at least one statistical significant repositioning event for H_2_O_2_, whereas two subjects demonstrated no significant alteration in CT organization for UVB (subjects 1, 2). More chromosomes were involved in change following H_2_O_2_ (ten CTs), than UVB (nine CTs). Also certain chromosomes seemed to be frequently repositioned in multiple subjects following H_2_O_2_ exposure (e.g. chromosomes 6, 8, and 10); in contrast a less consistent picture emerges from UVB exposed cells (Table 1). Furthermore, this table demonstrates variability in the types of movement observed following exposure to H_2_O_2_, in comparison to a more consistent predominant type in UVB. Based on the histograms produced from the radial analysis, movement of CTs was classified into the following categories: a) interior to less interior, b) interior to intermediate, c) interior to periphery, and d) intermediate to periphery. Fig. 6 (A–D) displays examples for all of the classified categories of CT movement observed in exposed cultures versus unexposed lymphocytes.

**Table 1:** Statistically significant events of CT repositioning following exposure to H_2_O_2_ and UVB compared to control unexposed lymphocytes.

Subject Number	H_2_O_2_ Chromosome	Movement	UVB Chromosome	Movement
1	6	Interior to Intermediate	-	-
1	8	Interior to Intermediate	-	-
1	10	Interior to Intermediate	-	-
2	8	Interior to Intermediate	-	-
3	7	Intermediate to Periphery	19	Less interior localization
3	8	Interior to Periphery	-	-
4	10	Less interior localization	15	Less interior localization
4	-	-	19	Less interior localization
5	4	Interior to Periphery	10	Less interior localization
5	6	Interior to Intermediate	12	Less interior localization
5	10	Less interior localization	15	Less interior localization
5	12	Less interior localization	17	Less interior localization
5	14	Less interior localization	22	Less interior localization
5	17	Less interior localization	X	Less interior localization
5	19	Less interior localization	-	-
5	X	Interior to Periphery	-	-
6	6	Interior to Intermediate	4	Interior to Intermediate
6	8	Interior to Periphery	8	Less interior localization
6	10	Interior to Intermediate	-	-

Table 1 includes chromosomes that demonstrated statistically significant events of repositioning (p<0.05) after exposure to H_2_O_2_ or UVB, compared to unexposed lymphocytes in the same subject. The percentage of fluorescence in each shell was compared between control and exposed lymphocytes and the χ^2^ goodness of fit test deemed whether significant events of CT repositioning occurred. Results are presented on a per subject basis and include the chromosomes involved and the altered positioning observed in the exposed lymphocytes compared to unexposed lymphocytes. The hyphen (-) denotes no significant event of repositioning observed for the exposure condition.

### Global and hierarchical distribution of all 24 human chromosomes in unexposed and exposed lymphocytes

**Fig 7:**
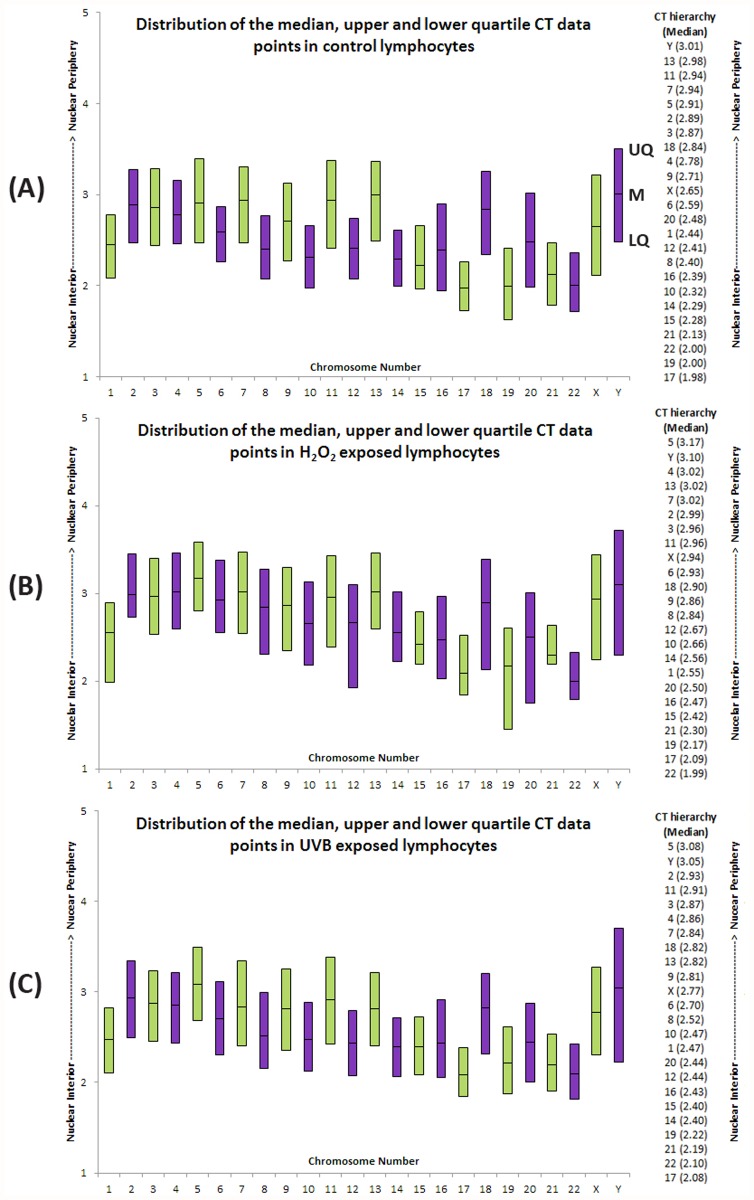
Hierarchical organization of all 24 human chromosomes and distribution of the median, upper and lower quartile CT data points in control H_2_O_2_ and UVB exposed lymphocytes. Box-plot representation of the distribution of all 24 chromosomes within lymphocytes in all six subjects. Panels A, B and C depict the distribution in control, H_2_O_2_, and UVB exposed lymphocytes, respectively. Each histographic bar represents the upper quartile, median, and lower quartile for each CT position from the nuclear interior to the nuclear periphery (Y axis). Panel A, displays the points that correspond to the upper quartile (UQ), median (M) and lower quartile (LQ) on the Y chromosome. Odd numbered chromosomes and chromosome X are represented by green bars, whereas even chromosomes and chromosome Y are represented by purple bars. The column of numbers to the right of the box-plot displays the hierarchical organization of CTs determined from the median CT data for the corresponding exposure conditions. The column of CTs are ordered from the nuclear interior (bottom) toward the nuclear periphery (top). Numbers in parentheses represents the mean median value for each CT (lower median values indicate closer proximity to the nuclear interior). Note, data for all CTs is based on measurements taken in 600 cells (n = 6) with the exception of the Y chromosome, which is obtained from 200 data points (n = 2).

In addition to measuring the radial distribution of fluorescence signal within each of the five rings (Figs. 2–4), the software also compresses the CT into a single number (median) that represents the midpoint of the CT. This median value can be utilized to hierarchically order the CT distribution from the nuclear interior to the nuclear periphery [41]. The average hierarchical order of all CTs in each condition is presented in Fig. 7 for the six subjects. In addition to the median, the average upper and lower quartile distribution of fluorescence for each CT and condition for the six subjects is displayed as a box-plot to allow a visual comparison of the global distribution of these points within the nucleus (Fig. 7 A-C). The length of the boxes demonstrates the variations observed in position of the CT for these points (median, upper, and lower quartile) among a population of cells and different exposures in six subjects. Shorter boxes suggest the CT is more constrained (less variation in CT position between cells and subjects), with larger boxes suggesting more variation in CT position between cells and subjects. Visual comparisons of the median reference point, relative length and position of the boxes between the various conditions reveal similar distribution patterns in the unexposed and UVB exposed lymphocytes (Fig. 7A and 7C). This distribution in the unexposed lymphocytes can be characterized as relatively constrained, and appears to be maintained following UVB exposure although a tighter clustering of these CTs points in the nucleus is observed (Fig. 7C). A different picture emerges from the H_2_O_2_ exposed CT distribution (Fig. 7B); with CTs displaying a larger distribution and more peripheral localization (e.g. CT 12 and 19, suggesting greater variation in CT position) compared to unexposed lymphocytes (Fig. 7A). The data depict a specific trend for chromosomes located closer to the interior of the nucleus. In both control and treated lymphocytes, chromosomes 17, 19, 21, and 22 seem to compile the core of the territories that are always found at the innermost region of the nucleus with chromosome 15 in close proximity for all conditions. Chromosomes 17, 19, and 22 are amongst the most gene dense, with chromosomes 15 and 21 less so [42]. Following that, the interior to intermediate areas of the nucleus are being accommodated by a mixture of medium to small sized chromosomes (e.g. chromosomes 6, 8, 9, 10, and 12) with the exception of chromosome 1. The emerging picture for the territories mostly occupying the intermediate to peripheral space, includes predominantly the largest of the chromosomes 2, 3, 4, 5, 7, 11, and 13. In terms of the sex chromosomes in all conditions X is more internally located compared to Y.

## Discussion

Historically, an inherent connection exists between CTs and DNA damage. One of the first pioneering experiments that re-discovered the concept of CTs and their non-random positioning involved the induction of DNA damage. This study utilized a small laser microbeam in a specific part of a Chinese hamster nucleus and identified that as a result, only a few chromosomes were damaged [43]. This finding was fundamental for verifying non-random organization of the interphase nucleus. The purpose of our study was to assess the effect of genotoxicity by two different agents in human lymphocytes in terms of the topology of chromosomes. Our study clearly demonstrates a reproducible CT organization in human lymphocytes between subjects; and provides evidence of chromosome repositioning following in-vitro exposure to genotoxicants. Despite genotoxicant exposure occurring 71 hours prior to harvesting of cells, measurable genotoxicity was induced in lymphocytes cultured in the presence of H_2_O_2_ and UVB. Specifically, a reduction in cellular proliferation was observed in H_2_O_2_ and UVB exposed lymphocytes (62% and 40.5%, respectively). Mitotic proliferation is frequently utilized in the assessment of genotoxity, and the findings of this study are similar to previously published studies [44–46]. The marked reduction in cellular proliferation observed in this study is likely due to altered cell cycling and/or a consequence of apoptosis as the result of genotoxicity [47]. However, it should be noted that measurement of the levels of apoptosis was not assessed in the current study to determine whether this was a contributing factor to the decreased cell proliferation observed. Furthermore, additional findings of cytogenetically visible chromosome aberrations provide further evidence of genotoxicity. The most common aberrations observed included pulverized chromosomes and chromosome or chromatid breaks. Overall, more aberrations were observed in the H_2_O_2_ exposed cultures however; UVB induced a wider variety of aberrations. The presence of cytogenetic chromosomal aberrations observed in this study has demonstrated that a proportion of cells contained DNA damage. Furthermore, these cells were able to progress through the cell cycle and initiate mitosis (at least up to the metaphase stage) despite the presence of persistent DNA damage. It should be noted that karyotyping can only detect structural aberrations that are >5Mb in size. Therefore, the percentage of abnormal metaphase spreads (around 8–9%), is likely to be an underrepresentation of total DNA damage due to the inability to identify smaller chromosomal aberrations. The results from the karyotype analysis are in line with previously published studies for these agents [48–50]. Genotoxicity observed was measurable over 71 hours following exposure to the genotoxic agents. Thus, it is important to realize that the lymphocyte cell population studied conceivably consists of a heterogeneous population of cells including: 1) cells in which exposure to the genotoxic agents did not induce any DNA damage; 2) cells in which DNA damage was induced, but successfully repaired; 3) cells undergoing apoptosis, following DNA damage and unsuccessful repair; and 4) cells in which apoptosis was evaded following DNA damage, but the DNA damage was not successfully repaired (e.g. karyotype aberrations observed).

We report for the first time a comprehensive picture of the genome organization of all 24 chromosomes in lymphocytes from more than one individual. In contrast to previous reports where one donor was used, six karyotypically normal subjects participated in this study [9,10,51]. The findings of this study demonstrated remarkable reproducibility of CT organization between subjects for 17 of the 24 investigated chromosomes. A handful of differences in the radial distribution of six chromosomes were identified (CTs 3, 10, 12, 13, 15, 17 and 19). It should be noted that differences often involved a single chromosome in a single subject with differences in organization between two other subjects. However, CT10 seems to have bimodal pattern of organization. Overall subject 6 demonstrated more differences, with a different CT position for 3 chromosomes compared to a single subject and a significantly less internal localization of CT19 compared to all subjects. Observations of inter-individual reproducibility of specific CTs (albeit a small sample size), demonstrates the importance of this organization. Our results confirm that chromatin inside the nucleus is a dynamic entity and in constant motion [52]. Thus, all chromosomes exhibited some variation in positioning; however, some were more defined than others with over 50% of CTs occupying distinct non-random positions. With the exception of chromosome 1, the medium (6, 8, 10, 12, 14, 15) and smaller-sized chromosomes (16, 17, 19, 20, 21, 22) consistently exhibited non-random positions in all subjects. In contrast CTs, 2, 3, 4, 5, 7, 13, 18, and Y seem to have a less defined (random) position in the nucleus (p>0.05). Furthermore, these results suggest that CT organization, at least for certain chromosomes, could be utilized as part of a battery of genotoxicity assays to measure nuclear health and monitor the level of DNA damage and/or DNA repair response.

We also purport repositioning of specific CTs following exposure to genotoxic agents (H_2_O_2_ and/or UVB). CT repositioning was observed in all subjects for at least one chromosome, three days following exposure to the genotoxicants. Overall, more repositioning was observed cumulatively following exposure to H_2_O_2_ than UVB (18 vs. 11 events). Seven CTs were involved in a statistically significant alteration of their position that were common in both H_2_O_2_ and UVB treated cells (4, 8, 10, 12, 17, 19, and X), whereas repositioning of chromosomes 6, 7, and 14 was exclusive to H_2_O_2_ and chromosomes 15 and 22 to UVB exposure. Of note, is the observation, that several chromosomes were frequently repositioned in multiple individuals following H_2_O_2_ exposure and to a lesser extent UVB exposure (chromosomes 6, 8, 10, and chromosomes 15, and 19 respectively). Inter-individual variability of CT repositioning following genotoxicant exposure was observed; with some subjects exhibiting less change (e.g. subjects 1 and 2) than others (e.g. subject 5). This inter-individual difference warrants further investigation and could be due to any number or combination of endogenous or exogenous factors (e.g. sensitivity to genotoxic agents, efficiency of DNA damage recognition and repair, age, diet, environment, stress, exercise, medication, and pathology). However, such studies in humans are notoriously difficult to design and control and are beyond the scope of this study. Another emerging feature was the greater versatility in the types of CT repositioning following exposure to H_2_O_2_ compared to UVB. The predominant positional alteration of CTs after UVB damage was a more constrained movement (less interior distribution) compared to H_2_O_2_ that appeared to possess a greater degree of movement or potentially chromatin decondensation. In the current study, all the repositioning events observed were peripheral movements, with no significant reciprocal internal repositioning of CTs. It is possible that one or more CTs adopted a smaller-scale internal repositioning event in response to the peripheral repositioning of other CTs that was not detectable in our current system. Whether the repositioning events observed following exposure to genotoxicants are the result of inter-individual variation or reflect a random disruption of genome organization remains to be elucidated. Given that the experiment was not repeated in the same individuals it was not possible to directly test these hypotheses. However, our preliminary data has identified a handful of chromosomes that were more susceptible to repositioning, suggesting that certain regions of the genome could be preferentially involved. It is clear that these hypotheses may not be mutually exclusive and warrant further investigation in future studies.

Mehta et al. recently used a similar methodology for all human chromosomes, examining whether CTs are repositioned as a result of genotoxic exposure including two fibroblast cell lines [53]. This study reported that certain CTs repositioned towards the nuclear periphery (CTs 17, 19, and 20), whereas others moved towards the nuclear interior (CTs 12 and 15) [53]. Despite the difference in the shape and size of fibroblasts and lymphocytes, they broadly have a similar CT organization as reported by Boyle et al. [10], with CTs 1, 16, 17, and 22 being located toward the nuclear interior and CTs 2, 13, and 18 being located more toward the nuclear periphery. Both our data and those of Mehta et al. [53] for unexposed cells are largely consistent with those of Boyle et al. [10]. Boyle et al. [10], classified CTs into four groups based on whether there was a predominance of fluorescence in the following: 1) nuclear interior (CTs 1, 17, 16, 22, and 19); 2) no significant bias toward the interior or periphery (5, 21, 15, 10, 6, 1 and 4); 3) less significant peripheral distribution (12, 9, X, Y and 20); and 4) nuclear periphery (7, 3, 13, 2, 8, 18, 11, and 4). Comparing this data with the radial hierarchy of CTs in this study from six subjects demonstrates very similar groupings of CTs (Fig. 7). The largest differences in the radial distribution between the studies are for CTs 8, Y and 5, with CT 8 being more internally localized in this study, whereas CTs Y and 5 were more peripherally localized in this study compared to Boyle et al. [10]. Five other chromosomes in this study showed a small difference in localization, with CTs 1, 6 and 16 being slightly more peripherally localized and CTs 12 and 21 being slightly more internally localized in the current study compared to that of Boyle et al. [10]. The relatively small variations observed between the two studies are reassuring, suggesting reproducibility between different erosion analysis software and different methods to assess the hierarchical radial organization of CTs. Additionally, it is important to note that one of the major strengths of the current study is the inclusion of multiple subjects rather than a single individual as examined in the Boyle et al. study [10]. When looking at all participants in our study, 12 different CTs (4, 6, 7, 8, 10, 12, 14, 15, 17, 19, 22, and X) were involved in a topological alteration (p<0.05) following damage from H_2_O_2_ and UVB. CT repositioning for chromosomes 12, 17, and 19 were common with the Mehta study [53]. The current study included six subjects also allowing the inter-individual variability and reproducibility of repositioning following genotoxicant exposure to be evaluated and also to identify common CTs (4, 8, 10, 12, 17, 19, and X) that were repositioned as a result of H_2_O_2_ and UVB damage. Our data suggests that both gene rich (e.g. 12, 17, and 19) and gene poor (e.g. 4, and 8) chromosomes [42] were associated with CT repositioning, compared to only gene rich chromosomes as observed in the Mehta study [53]. Several factors could account for the differences observed between the two studies: cell type, culture conditions, genotoxic agents, exposure conditions and lack of assessment of variability and reproducibility between subjects as performed in the current study.

Repositioning of CTs as a result of DNA damage has also been observed when HeLa cells were irradiated with α particles to initiate linear double strand breaks (DSBs) and distortions of the track morphology of CTs through γ-H2AX staining suggested movement of CTs [54]. Other studies using a similar methodology to track repair foci, post irradiation damage yielded mixed results in mammalian cells with little (photosensitized cells) or large distance movement (osteosarcoma and HeLa cells) [52]. The bodyguard hypothesis proposes preferential damage of DNA occurs in the peripheral heterochromatin to protect the euchromatin in the nuclear interior. However, localization of repair sites following H_2_O_2_ and UVC damage in fibrosarcoma cells toward the center of the nucleus in conjunction with the higher mutation rate seen in the internally located CT19 [55] argues against this hypothesis. Therefore, the nuclear center could be the preferred site for DNA damage and mutation with CTs located in the interior and intermediate region of the nucleus being preferentially repositioned with the exception of a single peripherally located CT7 following H_2_O_2_ exposure [37]. However, it is important to note that differences in nuclear localization of DNA damage and CT repositioning will likely depend on different types of damage, repair mechanisms, cell lines and growth conditions [52]. Moreover, we should not neglect that the spatial organization of chromosomes and genes is dynamic with repositioning occurring as result of gene expression, quiescence, senescence, mutations and diseases [21,27]. The movement of chromosome territories as result of DNA damage is not a surprise when one considers that chromatin is in constant relative motion during interphase and this motion fluctuates with ATP levels. Any constraint on the movement stems from the chromatin fiber itself, the nature of nucleoplasm and protein-protein interactions that tether loci to nuclear structures [52]. It appears that this movement of chromatin upon induction of a DNA lesion (e.g. DSB) is part of the general cascade of events that form the DDR of the cell to DNA damage. Evidence comes from the appearance of γ-H2AX molecules immediately after DSB through phosphorylation of H2AX histones at serine 139. This appears to be a critical and evolutionary conserved mechanism for chromatin reorganization that allows accessibility of repair factors to the damaged site [8,37]. The appearance of these molecules seems to be more correlated with gene rich chromosomes [53] or euchromatic regions [8]. Further evidence validating movement being part of the DDR response comes from yeast, where mutated upstream components of DDR resulted in DSBs and loss of enhanced mobility [52]. In mammalian cells a similar lower mobility has been described when uncapped telomeres were misinterpreted for DSBs, in cells with a null ATM mutation (involved in DSB motion) [56].

Our results demonstrate that CTs following H_2_O_2_ damage had a greater variation in nuclear distribution compared to control and UVB exposed cells. The findings in this study could potentially indicate more decondensed chromatin, mobility and plasticity of CTs following H_2_O_2_ exposure and warrants further investigation. Chromatin typically decondenses around sites of DNA damage to allow access of the repair machinery or to reduce the error risk during homologous recombination repair [8,57]. Additionally, differences in CT repositioning following exposure to genotoxic agents could simply be a reflection of the different way that the lesion is sensed and subsequently repaired [52]. It is clear that different repair rates exist following UV irradiation in mammalian cells with repair occurring faster in GC rich, followed by AT rich and then heterochromatic regions of the genome [38]. It seems plausible that the differences observed in the distribution of CTs within the nucleus between the two genotoxic agents likely reflects differences in the amount of damage induced; different mechanisms of DNA damage and DDR that occur in response to the different agents. Furthermore, it seems likely that subject-specific differences in susceptibility to DNA damage, DNA damage recognition, fidelity and efficiency of DDR will exist. This could in part, explain some of the inter-individual differences in CT repositioning observed in this study (e.g. number of events and CTs involved).

In terms of the hierarchical radial organization of lymphocytes reflects more of a gene-density correlation which is in agreement with previous findings [9,18,51,58]. Gene rich chromosomes (e.g. chromosomes 1, 15, 16, 17, 19, and 22) were preferentially located towards the nuclear interior, whereas gene poor chromosomes (e.g. 2, 3, 4, 5, 13, and 18) were located more towards the periphery. Certain chromosomes (e.g. 15, 16, 17, and 22) contain segmental duplications that are associated with regions of chromosome instability or evolutionary rearrangement [59,60]. Therefore, one possible explanation for their topological arrangement is for protection. The nuclear center is postulated to be an active transcription site, which plays a role in the maintenance of genome stability [37]. The sex chromosomes were localized in the intermediate/peripheral region of the nucleus. Our data agree with previously published studies, with the X chromosome demonstrating a more interior localization in comparison to the Y chromosome [10].

Our preliminary findings need to be expanded to a larger study, but suggest that certain CTs maintain a reproducible non-random organization between individuals and that certain CTs were frequently involved in genotoxic specific repositioning events following DNA damage. The emerging picture supports the compartmentalization of chromatin; thus, nuclear organization may influence the repair process. Recent evidence from yeast suggests that overlapping nuclear territories repair more efficiently than sequences located in spatially distant territories [61]. Whether it is the recombinational efficiency or a transcriptional surge that drives the repositioning of CTs warrants further investigation. Current data between repair and transcription with chromatin mobility seems to be correlative, and experiments that would address in real time the transcriptional output during a repositioning event are needed to provide more direct evidence [52].

## Conclusion

In summary, current evidence suggests organization of chromosomes likely plays an important role in the maintenance of genome integrity. Radial organization of chromosomes has the potential to be utilized as a powerful research and clinical diagnostic tool. In order to do so it is essential to evaluate and identify targets which are non-randomly organized in a “healthy state” and repositioned in “disease or damaged states”. Additionally, future studies should investigate CT repositioning at different time points and longer exposures to determine whether repositioning of CTs are transient or longer term effects. Temporal studies assessing the effect of genotoxic agents and CT repositioning with additional assays to measure DNA damage (e.g. comet, micronuclei, TUNEL) in combination with molecular studies to measure DNA repair will provide important information regarding: 1) which regions are more prone to damage as the result of specific agents; 2) DNA damage induced; 3) repair responses elicited; and 3) how CTs are repositioned in response to genotoxicity.
